# Meta regression analysis to indirectly compare dalteparin to enoxaparin for the prevention of venous thromboembolic events following total hip replacement

**DOI:** 10.1186/1477-9560-9-3

**Published:** 2011-01-27

**Authors:** George Dranitsaris, Valentina Jelincic, Yoonhee Choe

**Affiliations:** 1 Augmentium Pharma Consulting, Toronto, Canada and Eisai Inc., Woodcliff Lake, New Jersey, USA

## Abstract

**Background:**

Patients undergoing elective total hip replacement (THR) surgery are at an increased risk for venous thromboembolic events (VTEs). Dalteparin and enoxaparin are recommended as thromboprophylaxis for at least 10 days in these patients. Even though both agents have proven clinical effectiveness through placebo controlled studies, there have been no head to head trials to assess comparative effectiveness. Indirect statistical techniques were used to compare safety and efficacy between dalteparin and enoxaparin following THR surgery.

**Methods:**

A literature search was conducted from January 1980 to November 2009 for randomized trials evaluating dalteparin or enoxaparin prophylaxis in THR patients. In trials where a common control was used (e.g. placebo), indirect statistical comparisons between dalteparin and enoxaparin were performed using meta regression analysis with active drug as the primary independent variable.

**Results:**

A total of nine placebo controlled enoxaparin (n = 5) and dalteparin (n = 4) trials met the inclusion criteria. THR patients treated with enoxaparin or dalteparin had a 50% VTE risk reduction compared to the placebo control (RR = 0.50, p < 0.001). This benefit was achieved without a significant increase in the risk for major bleeds (RR = 1.19, p = 0.76), heparin induced thrombocytopenia (HIT) (RR = 1.13, p = 0.83) or death (RR = 0.72, p = 0.59). The indirect comparison was not able to find significant differences between enoxaparin and dalteparin in terms of VTEs (p = 0.36), major bleeds (p = 0.45), HIT (p = 0.48) and death (p = 0.86).

**Conclusions:**

The findings suggested comparable safety and efficacy between dalteparin and enoxaparin in TKR patients. Therefore, treatment decisions should be based on other considerations, such as patient or physician preference, ease of administration and cost.

## Introduction

Deep vein thrombosis (DVT) and pulmonary embolism (PE) are manifestations of venous thromboembolic events (VTE). The primary cause of a PE is a DVT. Following invasive procedures such as orthopedic surgery, patients are at risk for developing a DVT and subsequent PE.^1 ^This is a major clinical concern because PE can be fatal in 70% of cases, usually within the first few hours [1]. DVT and PE can also be common events in hospitalized patients. In one claims based study, it was determined that 0.64% (i.e. 32,193 cases) of all patients discharged from evaluated U.S. hospitals between 1998 to 2004 had DVT or PE as the primary discharge diagnosis, and 26,159 (0.52%) had DVT or PE as a secondary discharge diagnosis [2]. In the U.S., the high prevalence of VTE translates to a cost impact of approximately $2.9 billion annually [3].

To prevent VTE and avoid the associated health care costs, thromboprophylaxis following major orthopedic surgeries such as total hip replacement (THR) is standard practice. According to the American College of Chest Physicians (ACCP) guidelines, either a low molecular weight heparin (LMWH), or warfarin are recommended as thromboprophylaxis for at least 10 days in patients undergoing THR[4]. Extended prophylaxis for up to 35 days is also recommended in patients undergoing major orthopedic surgery, particularly for those at high-risk of DVTs [4]. The LMWH have some practical advantages over other agents. Anticoagulation therapy with warfarin or unfractionated heparin (UH) can be problematic in some patients because of the potential for drug-drug interactions and unpredictable anticoagulation levels secondary to liver dysfunction and poor patient nutrition [4,5]. In addition, there is a need to closely monitor patients receiving these alternative drugs.

Two of the most commonly used agents in the U.S. include enoxaparin and dalteparin. Despite being available for clinical use since the early 1990s, there are no large head to head randomized phase III studies between dalteparin and enoxaparin in patients undergoing major orthopedic surgeries such as THR. Physicians have been relying on "gut feeling" as opposed to empirical evidence that the products have comparable safety and efficacy. In the absence of a randomized trial, statistical methods can be used to indirectly evaluate two drugs. The advantages of using indirect statistical techniques to conduct comparative effectiveness evaluations are that they utilize the best available evidence to provide answers to questions that have not been addressed through a randomized trial. There are several example in the literature where indirect statistical analysis has been used to conduct comparative effectiveness research in diverse therapeutic areas such as kidney cancer and pulmonary arterial hypertension [6,7]. In this study, two unique statistical techniques were used to perform an indirect clinical comparison on the safety and efficacy between dalteparin and enoxaparin in patient undergoing THR surgery.

## Methods

### Literature Review and Meta Analysis of Randomized Trials

A computer literature search of PubMed, Embase, the Cochrane Database and Google Scholar was conducted from January 1980 to November 2009 for randomized trials evaluating dalteparin and enoxaparin for the prevention of VTEs in patients undergoing THR. Search terms consisted of {dalteparin or enoxaparin}, AND {prophylaxis} AND {THR}, OR {orthopedic surgery}, AND {randomized clinical trial}".

The inclusion criteria for trial acceptance consisted of the following:

• The trial must have utilized a parallel group design. Cross over trials were excluded given the inherent contamination from latent treatment effects in their overall design.

• The trial population must have consisted of patients undergoing THR.

• Must have enrolled patients greater than 18 years of age.

• The control arm in accepted trials must have been placebo, UH or warfarin.

• Accepted trials must have evaluated the clinically appropriate doses of dalteparin or enoxaparin in at least one of the trial arms

• Trials must have been a randomized comparison with at least 25 patients enrolled into each arm.

Once the trials meeting the inclusion criteria were identified, the following data were extracted: baseline patient information, indication, sample size in each group, drug and dosage, duration of therapy, study duration, definition of primary and secondary endpoints, primary and secondary results. Other data extracted from the accepted THR trials consisted of number of proximal, distal, symptomatic DVTs and PE in each group. Safety data consisted of bleeding events (minor and major), heparin induced thrombocytopenia (HIT), number of withdrawals caused by adverse drug reaction and all cause mortality in each of the study groups.

All of the study outcomes were presented as binary endpoints (e.g. VTE rates, major bleeding events) and were combined using a fixed or random effects model in cases of significant heterogeneity [8]. Random-effects meta-analysis assumes that the effect of the intervention varies across studies. When significant between-study variation is present, the 95%CI for the summary measure tends to be larger with a random-effects model. Treatment effects from individual trials were also presented as Forest Plots.

Statistical heterogeneity between studies was assessed by both the Q-statistic and the I^2 ^test statistic [9]. Briefly, the I^2 ^statistic measures the proportion of variance across studies due to heterogeneity. It is considered to be a superior measure of study heterogeneity than the Q-statistic because the latter is often underpowered when evaluating homogeneity in meta analyses. The p-values associated with the Q-statistic (chi-square with k-1 degrees of freedom, where K is the number of studies) were also reported. In situations where the Q-statistic was statistically significant or the I^2 ^statistic was greater than 25%, a random effects meta analysis model was used as described earlier. Publication bias was assessed through an evaluation of funnel plots and by the method proposed by Egger, which provides a significant p-value when publication bias may be present [10,11].

### Indirect Statistical Comparisons between Drugs

For each patient population, the indirect statistical assessment between dalteparin and enoxaparin was performed using two approaches. The first indirect method was a meta regression analysis on the primary clinical outcomes and on adverse event rates reported in the trials. A meta regression analysis is an appropriate method for conducting an indirect comparison in cases where trials evaluating the drugs of interest used a common comparator. Therefore, indirect comparisons between dalteparin and enoxaparin were performed with those trials that had the same control group. Separate analyses were performed using placebo and UH as the common control. Active drug (dalteparin and enoxaparin) was the independent variable in the regression model [7,12]. Other independent variables considered in the models included duration of therapy, treatment schedule (pre vs. post surgery initiation), geographic region where the study was conducted (i.e. North America vs. Europe vs. global) and year of publication. All of these analyses were performed using Stata, V 9.0 (Stata Corp., College Station, Texas, USA).

Indirect treatment comparisons were also performed using the method of Bucher and colleagues, which partly maintains the benefits of randomization on the effect size [13,14]. Briefly, this is a simple method for an adjusted analysis, in which the indirect comparison of A and B is adjusted according to the results of the direct comparisons with a common intervention - C. Let *lnOR*_*AC *_denote log odds ratio of *A*_*1 *_versus *C*_*1 *_in trial 1, and *lnOR*_*BC *_denote log odds ratio of *B*_*2 *_versus *C*_*2 *_in trial 2. The log odds ratio of the adjusted indirect comparison of *A *and *B (lnOR'*_*AB*_*) *can then be estimated by:

• *lnOR'AB *= *lnOR*_*AC *_*- lnOR*_*BC*_

• The standard error would be: *SE(lnOR'*_*AB*_*) = *√ *[SE(lnOR*_*AC*_*)*^*2 *^*+ SE(lnOR*_*BC*_*)*^*2*^*]*.

Although an odds ratio is used in the above equations, this adjusted method may also be used when the relative efficacy is measured as a relative risk, risk difference or mean difference. Empirical evidence indicates that results of adjusted indirect comparison are usually, but not always, similar to those of direct head-to-head trials [15].

It is important to make a distinction between the two methods for indirectly comparing dalteparin to enoxaparin. In the meta regression approach, the evaluation is between a LWMH (dalteparin and enoxaparin) vs. a common control (i.e. placebo or UH) and the effect measure is expressed as a relative risk difference between drugs. In contrast, the method of Bucher et al., uses a common comparator (e.g. placebo) to statistically link the two treatments. As a result, the generated outcome is an effect measure (RR in this case) comparing enoxaparin to dalteparin with an associated p-value for statistical significance [13,14].

## Results

A total of 150 citations were identified and reviewed. A total of 17 randomized trials meeting the inclusion criteria were appropriate for the statistical pooling exercise. Reasons for study rejection included duplicate publications, no active comparator, comparator other than placebo, UH or warfarin, less than 25 patients enrolled into each trial arm, dose finding study and patient population were non-THR. In 16 of the trials, the two common comparators to the LMWHs were either placebo or UH. There were no studies where warfarin was a common control for both drugs (Table 1).

**Table 1:** Randomized trials comparing dalteparin or enoxaparin in THR patients

Study	Sample Size (n)	Study Drug vs. Control	Results (n)	Major Bleeds (n)	HIT (n)	Deaths (n)
Dechavanne,1989 [17]	D1 = 42 D2 = 41 H = 41	D - 2500 IU SC 2 h pre-op, then q 12 h post-op × 10-13 days	DVT (all) D1 - 2 D2 - 3 H - 4		D1 - 0 D2 - 0 H - 0	
		D - 2500 IU SC 2 h pre-op, then q 12 h post-op × 48 h, then 5000 IU q AM × 10-13 days	DVT (proximal) D1 - 1 D2 - 1 H - 3			
		H - 5000 IU SC 2 h pre-op, then bid × 2 days post-op, then H dosage was adjusted according to aPTT × 10-13 days				

Tørholm, 1991 [16]	D = 60 P = 60	D - 2500 IU SC 2 h pre-op and 12 h post-op, then 5000 IU qAM × 6 days	DVT D - 9 P - 19			D - 1 P - 0
		P - 2 h pre-op and 12 h post-op, then qAM × 6 days	PE D - 0 P - 1			

Dahl, 1997 [19]	D = 134 P = 131	D - 5000 IU SC evening before op and qPM × 7 days, then 5000 IU SC qPM × 28 days (±2 days)	DVT within first 7 days D - 4 P - 3		D - 0 P - 0	D - 0 P - 0
		P - 5000 IU SC evening before op and qPM × 7 days, then placebo × 28 days (±2 days)	DVT within 35 days D - 22 P - 23			
			PE within 35 days D - 0 P - 1			

Lassen, 1998 [20]	D = 140 P = 141	D - 5000 IU SC qd, starting 12 h pre-op and × 7 days post-op; then 5000 IU qd × 28 days	DVT (all) D - 5 P - 12	D - 0 P - 1	D - 0 P - 1	
		P - 5000 IU SC qd, starting 12 h pre-op and × 7 days post-op; then placebo × 28 days	DVT (proximal) D - 1 P - 5			
			PE D - 0 P - 0			

Hull, 2000a [21]	D1 = 504 D2 = 494 W = 500	D1 (pre-op) - 2500 IU SC within 2 h before surgery, then 2500 IUSC 4 or more h after surgery, then 5000 IU SC qAM × 6 days (±2)	DVT (all) D1 - 36 D2 - 44 W - 81	First 8 days D1 - 44 D2 - 32 W - 22	D1 - 6 D2 - 4 W - 10	D1 - 2 D2 - 0 W - 2
		D2 (post-op) - Placebo within 2 h before surgery, then 2500 IU SC 4 h or more after surgery, then 5000 IU SC qAM × 6 days (±2)	DVT (proximal) D1 - 3 D2 - 3 W - 11			
		W - 10 mg PO evening after surgery (5 mg if ≥ 70 years old), then qd dosing by nomogram × 6 days (±2)	PE D1 - 0 D2 - 0 W - 0			

Hull, 2000b [22]	D1 = 199 D2 = 190 W = 180	D (pre-op) - 2500 IU SC within 2 h before surgery, then 2500 IU SC 4 or more h after surgery, then 5000 IU qAM in-hospital, then 5000 IU qd up to 35 days (±2)	DVT (all) out of hospital within first 35 days D1 - 8 D2 - 6 P - 14	Major bleeds out of hospital within first 35 days D1 - 0 D2 - 0 P - 0	D1 - 0 D2 - 0 P - 0	D1 - 0 D2 - 0 P - 1
		D (post-op) - 2500 IU SC 4 or more h after surgery, then 5000 IU qAM in-hospital; then 5000 IU qd up to 35 days (±2)	DVT (proximal) out of hospital within first 35 days D1 - 2 D2 - 1 P - 7			
		W - 10 mg PO evening after surgery (5 mg if ≥ 70 years old or < 57 kg), then qd dosing by nomogram in-hospital, then placebo up to 35 days (±2)	PE out of hospital within first 35 days D1 - 0 D2 - 0 P - 0			

Turpie, 1986 [23]	E = 50 P = 50	E - 30 mg SC bid, started 12-24 h post-op, then qd × 14 days or until discharge	DVT (all) E - 6 P - 21	E - 1 P - 2		E - 0 P - 1
		P - 30 mg placebo SC bid started 12-24 h post-op, then × 14 days or until discharge	DVT (proximal) E - 2 P - 10			
			PE E - 0 P - 0			

Planes, 1988 [24]	E = 124 H = 113	E - 40 mg SC starting 12 h (the day before surgery) pre-op, then qd × 14 days or until discharge	DVT (all) E - 15 H - 27	E - 2 H - 0		E - 0 H - 0
		H - 5000 IU SC starting 2 h (on the day of surgery) pre-op, then tid × 14 days or until discharge	DVT (proximal) E - 9 H - 20			
			PE E - 2 H - 3			

Levine, 1991 [25]	E = 333 H = 332	E - 30 mg SC bid, starting 12-24 h post-op, then bid × 14 days or until discharge	DVT (all) E - 57 H - 63	E - 11 H - 19	E - 0 H - 2	E - 0 H - 0
		H - 7500 IU SC bid, starting 12-24 h post-op, then bid × 14 days or until discharge	DVT (proximal) E - 16 H - 18			
			PE E - 0 H - **1**			

Colwell, 1994 [26]	E1 = 195 E2 = 205 H = 210	E1 - 30 mg SC q 12 h × 7 days E2 - 40 mg SC qdx 7 days H - 5000 IU SC q 8 h × 7 days	DVT (all) E1 - 9 E2 - 30 H - 24	E1 - 8 E2 - 3 H - 13	E1 - 7 E2 - 3 H - 5	E1 - 1 E2 - 0 H - 2
			DVT (proximal) E1 - 4 E2 - 8 H - 10			
			PE E - 0 H - 3			

Avikainen, 1995 [27]	E = 83 H = 84	E - 40 mg SC qd, starting 12 h pre-op, then qd × 10 days	DVT (all/proximal) E - 1 H - 4	E - 7 H - 7		
		H - 5000 IU SC bid, starting 2 h pre-op and 12 h post-op, then bid × 10 days	PE E - 0 H - 1			

Bergqvist,1996 [28]	E = 131 P = 131	E - 40 mg SC qd starting 12 h pre-opx 7-11 days then 40 mg SC qd post-op × 21 days	DVT (all) E - 21 P - 45		E - 1 P - 0	E - 0 P - 0
		E P - 40 mg SC qd starting 12 h pre-op × 7-11 days then placebo qd post-op up to 21 days	DVT (proximal) E - 8 P - 28			
			PE E - 0 P - 2			

Planes, 1996 [29]	E = 90 P = 89	E - 40 mg SC qd × 21 days P - qd × 21 days	DVT (all) E - 6 P - 17	E - 0 P - 0	E - 0 P - 0	E - 0 P - 0
			DVT (proximal) E - 5 P - 7			
			PE E - 0 P - 0			

Rader, 1998 [30]	E = 70 H = 56	E - 5000 IU SC PM pre-op and AM and PM op day, then post-op enoxaprin 40 mg SC qd × 13-21 days	DVT (all) E - 2 H - 1	E - 0 H - 0		
		H - 5000 IU SC PM pre-op and AM and PM op day, then 5000 IU SC tid × 3 days, then 7500 IU tid on 4th day post-op × 13-21 days	PE E - 0 H - 0			

Comp, 2001 [31]	E = 224 P = 211	E - 30 mg SC bid, starting 12-24 h post-op and continuing 7-10 days; then enoxaparin 40 mg SC qd × 18-21 days	DVT (all) E - 18 P - 49	E - 0 P - 0	E - 3 P - 2	
		P - enoxaparin 30 mg SC bid, starting 12-24 h post-op and continuing 7-10 days; then placebo qd × 18-21 days	DVT (proximal) E - 6 P - 27			
			PE E - 0 P - 1			

Senaran, 2006 [32]	E = 50 H = 50	E - 40 mg SC qd, starting 12 h pre-op × 7-10 days until discharge	DVT during hospitalization (first 7-10 days) E - 0 H - 2	E - 2 H - 0	E - 0 H - 0	E - 0 H - 0
		H - 5000 IU SC, starting q 8 h pre-op, then continued to 15,000 IU qd in 3 equal doses q 8 h × 7-10 days until discharge	DVT within 45 days after discharge E - 2 H - 0			
		* 45 day post discharge follow up	PE E - 0 H - 0			

Fuji, 2008 [33]	E1 = 104 E2 = 105 E3 = 107 P = 105	E1 - 20 mg SC qd, started 24-26 h post-op, then qd × 14 days	DVT (all) E1 - 21 E2 - 18 E3 - 27 P - 36	E1 - 1 E2 - 3 E3 - 2 P - 0		
		E2 - 20 mg SC bid, started 24-26 h post-op then qd × 14 days				
		E3 - 40 mg SC qd, started 24-26 h post-op, then qd × 14 days	DVT (proximal) E1 - 3 E2 - 3 E3 - 6 P - 9			
		P - qd × 14 days				
		*Follow-up at 90 days after surgery	PE E1 - 0 E2 - 0 E3 - 0 P - 0			

Abbreviations: D = dalteparin, E = enoxaparin, P = placebo, H = unfractionated heparin, SC = subcutaneously, W = warfarin, IV = intravenously, THR = total hip replacement, HIT = heparin induced thrombocytopenia, VTE = Venous thromboembolism, S = symptomatic, DVT = deep vein thrombosis, PE = pulmonary embolism

There were a total of 11 and 6 published randomized trials evaluating enoxaparin and dalteparin in this patient population (Table 1). Of the 11 enoxaparin trials, five were against placebo (one evaluating three enoxaparin arms), and six had UH in the control arm. With dalteparin, there were four placebo controlled trials, one was against warfarin and the remaining one was relative to UH. To apply the statistical techniques for the indirect comparison, a common control group is required. Therefore, separate analyses were conducted for the placebo and the UH controlled trials. This provided a total of nine placebo trials and seven against UH for the two drugs. A comparison using warfarin as a common control could not be conducted because there were no such trials for enoxaparin.

### Indirect Comparison using Placebo as the Common Control

**Figure 1:**
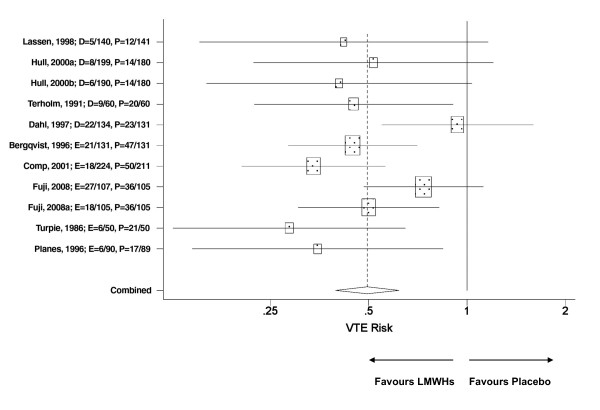
**Meta analysis on the relative risk of VTEs in placebo-controlled trials evaluating enoxaparin or dalteparin in THR patients**. The pooled VTE relative risk was significantly different between pharmacotherapy (dalteparin or enoxaparin) vs. placebo; p < 0.001. Test for heterogeneity: Chi^2 ^= 13.9, df = 10, p = 0.18, I^2 ^= 28.1%

**Figure 2:**
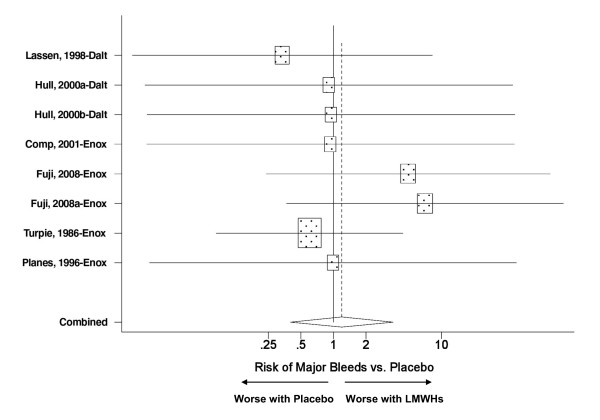
**Meta analysis on the relative risk for major bleeds in placebo-controlled trials evaluating enoxaparin or dalteparin in THR patients**. The pooled relative risk for major bleeds was not significantly different between pharmacotherapy (dalteparin or enoxaparin) vs. placebo; p = 0.76. Test for heterogeneity: Chi^2 ^= 3.34, df = 7, p = 0.85, I^2 ^= 0.0%

The nine placebo controlled trials provided a total of 11 treatment arms, six and five for enoxaparin and dalteparin respectively (Table 1). The VTE incidence data were pooled for all trials regardless of the LMWH used. The findings revealed that THR patients treated with a LMWH had a 50% reduction in the risk of a post surgical VTE (RR = 0.50, p < 0.001) - (Figure 1). Similarly, there was no indication that either LMWH was associated with neither an increase in the risk of major bleeds relative to placebo (Figure 2) nor any of the other relevant adverse events (Table 2).

**Table 2:** Summary of meta regression analysis on the risk of VTE and adverse events in THR patients

Outcome	LMWH (D or E) vs. Control	SE	P-Value	Impact on Risk
**VTE Risk in Placebo Trials**	**Relative Risk**			
Both drugs vs. placebo	0.50 (0.40 - 0.61)		<0.001	↓by 50%
RR difference between drugs (E vs. D)	0.23 (i.e. 12%)	0.25	0.36	NS
Region (vs. North American)				
European trial	0.33	0.49	0.50	NS
Global trial	0.20	0.58	0.73	NS
LMWH dosing^1^	-0.09	0.53	0.87	NS
LMWH treatment duration^2^	-0.21	0.31	0.51	NS
Year of publication	0.04	0.04	0.34	NS
Risk of Major Bleeds				
Both drugs vs. placebo	1.19(0.39 - 3.55)		0.76	NS
Risk difference between Drugs (E vs. D)	-0.94	1.25	0.45	NS
Risk of HIT				
Both drugs vs. placebo	1.13 (0.36 - 3.53)		0.83	NS
RR difference between Drugs (E vs. D)	-0.85	2.0	0.48	NS
Risk of Death				
Both drugs vs. placebo	0.72 (0.22-2.34)		0.59	NS
RR difference between Drugs (E vs. D)	0.22	1.28	0.86	NS
**VTE Risk in UH Trials**				
Both drugs vs. UH	0.83 (0.66-1.05)		0.12	NS
RR difference between drugs (E vs. D)	-0.24 (i.e. - 22%)	0.61	0.66	NS
Risk of Major Bleeds				
Both drugs vs. UH	0.70 (0.35-1.38)		0.30	NS
RR difference between Drugs (E vs. D)	NA			
Risk of HIT				
Both drugs vs. UH	0.61 (0.21-1.77)		0.36	NS
RR difference between Drugs (E vs. D)	0.56	1.53	0.71	NS
Risk of Death				
Both drugs vs. UH	0.59 (0.12-3.1)		0.53	NS
RR difference between Drugs (E vs. D)	NA			

Abbreviations: LMWH = low molecular weight heparin, D = dalteparin, E = enoxaparin, P = placebo, UH = unfractionated heparin, THR = total hip replacement, VTE = venous thromboembolic events, HIT = heparin induced thrombocytopenia, NS = not significant, DVT = deep vein thrombosis, PE = pulmonary embolism, NA = data not available for calculation.

^1^Pre vs. post surgical initiation. ^2^Short duration (i.e. < 15 days) vs. extended duration.

A meta regression model with "active drug" (enoxaparin vs. dalteparin) was then developed to compare VTE risk between drugs. The results were unable to find statistically significant difference between enoxaparin and dalteparin in the risk of VTEs in patients undergoing THR surgery (RR difference = 12%, p = 0.36). Similarly, there were no significant risk differences between LMWH dosing schedules (pre vs. post surgery initiation), treatment duration (< 15 days vs. extended therapy), trial location and year of publication (Table 2). The second phase of the meta regression analysis was an assessment of safety in terms of major bleeds, HIT and death. Comparing the two LMWH to placebo, there were no significant differences in any of these adverse events suggesting that both enoxaparin and dalteparin are safe to use in patients undergoing THR (Table 2).

### Indirect Comparison using Unfractionated Heparin as the Common Control

**Figure 3:**
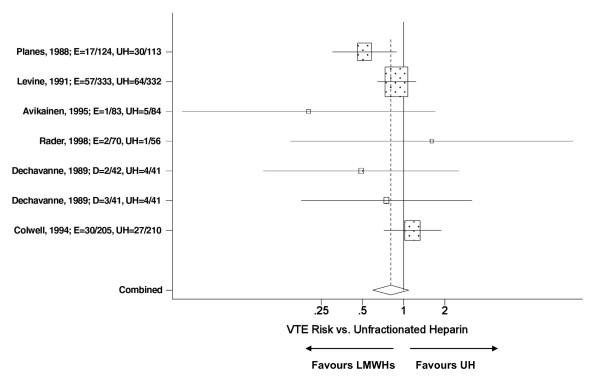
**Meta analysis on the relative risk VTEs in unfractionated heparin controlled trials evaluating enoxaparin or dalteparin in THR patients**. The pooled VTE relative risk was not significantly different between LMWHs (dalteparin or enoxaparin) vs. UH; p = 0.12. Test for heterogeneity: Chi^2 ^= 7.42, df = 6, p = 0.28, I^2 ^= 19.1%

There were a total of seven trials that compared enoxaparin or dalteparin to UH. The trials provided a total of 7 treatment arms suitable for meta analysis, five and two for enoxaparin and dalteparin respectively (Table 1). Regardless of the LMWH, the meta analysis identified a trend where patients randomized to a LMWH had a reduced risk for developing a VTE following THR (RR = 0.83, p = 0.12) compared to UH (Figure 3). When an analysis between drugs was undertaken, the meta regression analysis suggested comparable efficacy between enoxaparin and dalteparin, but a 22% relative advantage was noted in favour of dalteparin which did not reach statistical significance (Table 2).

A safety assessment was then undertaken using UH as the control. Due to the lack of data, we were unable to perform an indirect statistical comparison with respect to major bleeding events and death. However, there was no significant risk difference in the risk of HIT between dalteparin and enoxaparin (Table 2). In summary, the indirect statistical assessment of enoxaparin and dalteparin using meta regression analysis was unable to find any statistically significant differences between drugs with respect risk for VTEs, major bleeds, HIT and death regardless of the control used (Table 2). Therefore, these data support the comparative efficacy between enoxaparin and dalteparin in THR patients.

### Comparing Safety and Efficacy using the Method of Bucher et al. (1997)

The indirect method developed by Bucher and colleagues is one of the most cited approaches for performing indirect comparisons of randomized trials [13,14]. It was applied for the indirect assessment of enoxaparin vs. dalteparin with respect to VTE prevention and adverse events such major bleeds, HIT and death in patients undergoing THR surgery. As was previously suggested by the meta regression analysis, there was no evidence to suggest that one agent was better to another with respect to VTE prevention following THR surgery (Table 3). Similarly, there was no significant difference in the risk of major bleeds, HIT and death between the drugs as indicated by the 95%CIs for the RR which crossed the 1.0 threshold. Hence, these data imply that enoxaparin and dalteparin have comparable safety and efficacy when used to prevent VTEs in this high risk patient group.

**Table 3:** Summary of indirect statistical comparisons between dalteparin and enoxaparin using the method of Bucher et al., (1997)

Comparison	RR: E vs. D	(95%CI)	P-Value
**THR Patients: Placebo Trials**			
VTE	1.26	(0.85 - 1.88)	0.78
Major Bleeds	2.57	(0.22 - 29.8)	0.58
Thrombocytopenia	2.34	(0.22 - 24.4)	0.69
Death	0.80	(0.06 - 1.0)	0.90
**THR Patients: UH Trials**			
VTE	1.30	(0.42 - 4.1)	0.49
Major Bleeds	NA		
Thrombocytopenia	0.57	(0.03 - 11.4)	0.94
Death	NA	(0.03 - 17.9)	0.94

Abbreviations: RR = relative risk, D = dalteparin, E = enoxaparin, P = placebo, UH = unfractionated heparin, THR = total hip replacement, VTE = venous thromboembolic events, NA = data not available for calculation

### Testing of Publication Bias

The potential for publication bias was assessed. From the placebo-controlled THR trials, asymmetry in the funnel plot was detected (figure not shown) and the p-value from the Egger test (p = 0.19) indicated the possibility of publication bias. In contrast, there was no evidence to suggest publication bias with the UH-controlled THR trials (the Egger test, p = 0.44).

## Discussion

Dalteparin and enoxaparin have been available for DVT prophylaxis since the early 1990s. Several large well designed randomized trials demonstrated that these agents were at least equivalent to UH and superior to placebo for the prevention of VTEs follow major orthopedic surgery [16,17]. However, there have been few trials with sufficient sample size and statistical power to assess the noninferiority between the two drugs. Therefore, comparative outcomes in terms of safety and efficacy between dalteparin and enoxaparin has not been formally established [18]. Furthermore, the limited patent life of these drugs make it unlikely that a large noninferiority trial will be conducted to answer this important question. Therefore, physicians have tended to use "gut feeling" as opposed to formal evidence when assessing the comparative efficacy between the drugs. In the absence of direct comparative data, indirect statistical techniques are an appropriate alternative for providing the needed evidence [15]. In this study, two such indirect methods were used to compare the products for efficacy and safety as part of a larger meta analysis.

Our findings initially demonstrated that dalteparin and enoxaparin are highly effective in preventing DVTs following THR surgery. THR patients randomized to the LMWH arm of the trials were 50% less likely to develop a VTE during the study period compared to placebo. The drugs also had comparative effectiveness relative to UH in preventing VTEs in the same patient population. All of these benefits were achieved without an increase in the occurrence of major bleeds, HIT and overall mortality.

The evaluation was continued with a meta regression analysis which was unable to detect statistically significant differences between dalteparin and enoxaparin in terms of safety (i.e. HIT) and efficacy in THR patients. Given the available data from prospective randomized trials and the application of two indirect statistical techniques, it is reasonable to conclude that dalteparin provides comparative safety and efficacy to enoxaparin when used to prevent VTEs in patients undergoing THR surgery. To our knowledge, this is the first study to use indirect methods for comparing these two commonly used drugs.

The application of indirect statistical techniques in the absence of large head to head randomized trials is a reasonable approach to compare efficacy and safety between dalteparin and enoxaparin [14,15]. However, there are several limitations in this study that need to be acknowledged. All meta-analyses are affected by the quality of the studies analyzed. For that reason, we limited our review to prospective randomized trials with sufficient sample size. However given the nature of the intervention (i.e. UH), not all of the trials were double blinded. Regardless of the data source, our analysis was indirect and does not replace a well designed non-inferiority trial comparing the two drugs. Therefore, we must be aware of the potential biases associated with indirect comparisons. There were only seven randomized trials suitable for meta analysis in studies that using UH as the control. It must be acknowledged that this may have limited our statistical power, so the risk of a type II error (i.e. false negative) must be recognized as well as compromising our precision. The current analysis was not a true non-inferiority study because a pre-specified "*minimally clinically important difference*" in efficacy between the two drugs has not been established by regulatory authorities or the academic community. Some of the trials provided more that one two treatment arms for statistical analysis via meta regression. This may violate the independence assumption of regression modeling. Lastly, the failure to find statistically significant differences in the clinical endpoints between the two drugs through indirect methods does not confirm comparable efficacy. The only way to definitively answer this question is through a non-inferiority trial. However, it is a reasonable alternative in the absence of such a clinical trial.

## Conclusions

The findings of this meta analysis of prospective randomized trials suggest that dalteparin and enoxaparin are highly effective for VTE prophylaxis following THR and surgery. Keeping in the mind the caveats associated with cross trial statistical comparisons, our findings also suggested comparable safety and efficacy between dalteparin and enoxaparin. Therefore, treatment decision making should be based on patient preferences, ease of administration and cost considerations.

## Competing interests

Yoonhee Choe is an employee of the study sponsor.

## Authors' contributions

• GD: Study design, data review, data analysis and preparation of the manuscript.

• VJ: Literature search, data extraction and review and review of the manuscript.

• YC: Study design, data review and review of the final manuscript.

All authors have read and approved the final manuscript.
